# Rapid ungated myocardial perfusion cardiovascular magnetic resonance: preliminary diagnostic accuracy

**DOI:** 10.1186/1532-429X-15-26

**Published:** 2013-03-27

**Authors:** Alexis Harrison, Ganesh Adluru, Kavitha Damal, Akram M Shaaban, Brent Wilson, Daniel Kim, Chris McGann, Nassir F Marrouche, Edward V R DiBella

**Affiliations:** 1 Division of Cardiology, University of Utah, Salt Lake City, UT, USA; 2 CARMA, Department of Internal Medicine, Salt Lake City, UT, USA; 3 Utah Center for Advanced Imaging Research, Department of Radiology, University of Utah, Salt Lake City, UT, USA; 4 Department of Bioengineering, University of Utah, Salt Lake City, UT, USA; 5 Department of Radiology, University of Utah, Salt Lake City, UT, USA

**Keywords:** Gating, ECG, Ungated cardiac MR, Myocardial perfusion imaging, Cardiac perfusion, Contrast-enhanced MRI

## Abstract

**Background:**

Myocardial perfusion cardiovascular magnetic resonance (CMR) is a well-established method for detection of ischemic heart disease. However, ECG gating problems can result in image degradation and non-diagnostic scans, particularly in patients with arrhythmias.

**Methods:**

A turboFLASH saturation recovery pulse sequence was used without any ECG triggering. One saturation pulse followed by 4–5 slices of undersampled radial k-space images was acquired rapidly, on the order of 40–50 msec per image. The acquisition of the set of 4–5 slices was continuously repeated approximately 4 times per second. An iterative constrained reconstruction method was used to reconstruct the ungated images. The ungated perfusion images were post-processed into three different sets of images (ungated, self-gated to near systole, and self-gated to near diastole). To test the ungated approach and compare the different processing methods, 8 patients scheduled for coronary angiography underwent stress and rest perfusion imaging with the ungated acquisition. Six patients had a history of atrial fibrillation (AF). Three blinded readers assessed image quality and presence/absence of disease.

**Results:**

All 8 subjects successfully completed the perfusion CMR protocol and 7/8 underwent coronary angiography. Three patients were in atrial fibrillation during CMR. Overall, the CMR images were of high quality as assessed by the three readers. There was little difference in image quality between patients in AF compared to those in sinus rhythm (3.6±0.7 vs. 3.3±0.5). Stress/rest perfusion imaging showed normal perfusion in 4 patients, fixed perfusion defects in 2 patients, and reversible perfusion defects in 2 patients, corresponding with angiographic results. Pooled results from the independent readers gave a sensitivity of 0.92 (CI 0.65-0.99) and specificity of 0.92 (CI 0.65-0.99) for the detection of coronary artery disease using ungated perfusion imaging. The same sensitivity, and a specificity of 1 (CI 0.76-1), was achieved when the images were self-gated after acquisition into near systole or near diastole.

**Conclusions:**

Ungated radial dynamic perfusion CMR can give high quality imaging in patients in sinus rhythm and during atrial fibrillation. In this small cohort, high diagnostic accuracy was possible with this rapid perfusion imaging sequence. An ungated approach simplifies the acquisition and could expand the role of perfusion CMR to include patients with arrhythmia and those with gating problems.

## Background

Coronary artery disease (CAD) affects over 17 million people in the US [1] and is projected to increase in prevalence by 16% in the next 2 decades [2]. More than half of CAD diagnoses occur with a presentation of chest pain, in the absence of an acute myocardial infarction [1]. While the highest risk patients proceed to coronary angiography, there is an array of anatomical and functional noninvasive testing options to evaluate for coronary artery disease in the low and intermediate risk patients. Cardiovascular magnetic resonance (CMR) is an appealing choice as it lacks ionizing radiation and can provide a highly accurate comprehensive exam encompassing myocardial function, perfusion, viability, and coronary anatomy. CMR is becoming a more robust, clinically accepted perfusion modality as more evidence supports its excellent diagnostic accuracy [3,4]. A recent meta-analysis of 2100 patients and 26 studies reported a sensitivity of 89% and specificity of 80% for CMR perfusion [5].

Frequently acknowledged limitations to CMR include pacemakers and/or defibrillators and glomerular filtration rate (GFR) <30 ml/min [3]. As well, difficulties of gating in patients can also be extremely problematic and poor gating can significantly degrade the quality and usefulness of MR imaging. Many standard MR imaging sequences rely on even, repetitive timing of a cardiac cycle to obtain image information synchronized to the same phase of the cardiac cycle over multiple heartbeats and to eliminate cardiac motion. Without this precise timing or with very long pauses between triggers, datasets are distorted and can be uninterpretable. Magnetic field gradients, magnetohydrodynamic effects, and physical characteristics of the patient (scoliosis, barrel chest, and pericardial effusions) may degrade the ECG signal [6]. Furthermore, severe arrhythmia with variable R-R interval width may make many MR sequences unusable. This is particularly important in stress perfusion CMR which has a relatively short window of opportunity to acquire dynamic perfusion images of the first pass of contrast during maximal hyperemia. Highlighting this limitation, many perfusion CMR studies to date exclude patients with severe arrhythmia [7]–[13] or do not comment on the exclusion or inclusion of patients with arrhythmia [4,14,15].

We propose a novel acquisition method for perfusion CMR that is insensitive to poor gating and arrhythmia as it runs continuously without any gating signals. The images are acquired with a rapidity that allows for enough image collection to use the “beating” ungated images for diagnosis, or to sort the resulting dataset retrospectively and create effectively self-gated perfusion images. The aim of this study was to evaluate in an initial set of patients how well such an ungated or self-gated approach would work for detection of coronary artery disease.

## Methods

### Multi-slice “real-time” ungated data acquisition

A saturation recovery radial turboFLASH sequence was used with TR/TE=2.2/1.2 msec, on a 3 T magnet (Verio, Siemens Healthcare, Erlangen, Germany). Twenty or twenty-four radial k-space lines or rays in 4 subsets of 5–6 rays each were acquired for each slice. The starting angle was offset in each time frame, repeating every four time frames [16]. Five slices (four if 24 rays were used) were acquired after a single saturation pulse and a ~40 msec delay. Each image was acquired in 20-24*2.1-2.2 = 42-53 msec and was repeated without pause, approximately four times per second with no gating and during free-breathing. Thus each slice was acquired at various cardiac phases in each heartbeat.

### Image reconstruction

The images were reconstructed from undersampled radial k-space data with an iterative compressed sensing method using spatial and temporal total variation (TV) constraints [16,17]. The cost function *C* that is minimized is shown in the formula below where *E* is the forward modeling operator (Fourier transform and undersampling), *d* is the undersampled data, *m* is the image estimate. *D*_t_, *D*_x_ and *D*_y_ are gradient operators along time, *x* and *y* dimensions respectively. $C={\left\|\mathrm{Em}-d\right\|}_{2}^{2}+\alpha_{1}{\left\|\sqrt{D_{t}m^{2}+\varepsilon }\right\|}_{1}+\alpha_{2}{\left\|\sqrt{D_{x}m^{2}+D_{y}m^{2}+\varepsilon }\right\|}_{1}$

The two weighting factors, α_1_ for the temporal total variation and α_2_ for the spatial total variation constraints, were determined empirically for one of the ungated datasets and were fixed for all other datasets. Iterative gradient descent was used to minimize the cost function for each coil. The epsilon term in the cost function helps avoid singularities when minimizing *C* using the gradient descent method and was set to 1×10^-8^. The coil images were then combined by the square root of sum-of-squares.

**Figure 1:**
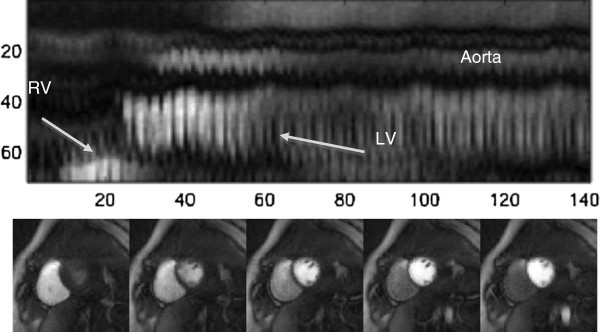
**Top: One cropped projection (from images like those on the bottom) from continuously acquired perfusion data, all frames.** Contrast onset in various structures can be observed, as well as cardiac and respiratory motion. This “navigator” corresponds to projecting (summing) along columns in the bottom images.

Figure 1 shows an example of the same projection of one slice acquired each time frame. This data is from a healthy volunteer imaged for the initial development of the approach (FOV=260 mm, 2.3×2.3×10 mm pixel size, 24 rays, 4 slices). The cropped projection corresponds to an area around the heart and shows a high frequency change due to changes in cardiac phase, a slower change due to respiration, and intensity changes due to contrast administration. The figure illustrates that it is likely feasible to obtain a gating signal from the projection data, as has been done for cine data with bandpass filters [18,19]. However, here we focus on a retrospective image-based gating method that is similar to a regional sum technique applied to self-gate cardiac cine data [6].

### Self-gating

**Figure 2:**
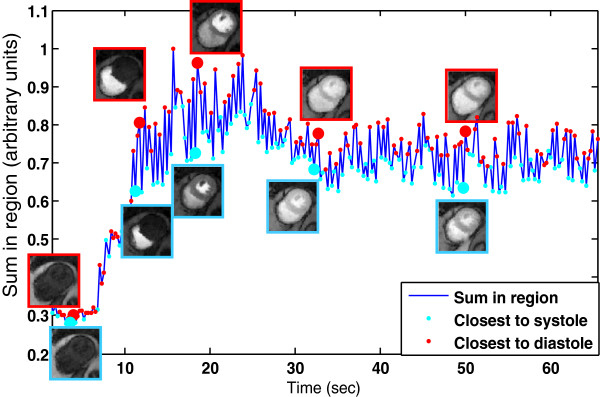
**Illustration of selected ungated time frames and the self-gating process in one slice.** The sum of a region around the heart in each time frame was used to generate a 1D self-gating signal, plotted in blue. The ungated images were then binned (self-gated) into near-diastole or near-systole if the 1D signal was a peak or a trough, respectively. The images above the plotted blue line are all near-diastole and their time points correspond to the large red circles. Near-systole images are shown below the plotted blue line and were acquired at the times marked by the large cyan circles.

After reconstruction, the ungated images were processed to self-gate into near-systole and near-diastole image sets. The self-gating method operated on a cropped region around the heart. This region was found automatically by first identifying the right ventricle from the time curves. A fixed size region encompassing the left ventricle (LV) and right ventricle (RV), centered on the RV, was then used as the cropped region. Summing up the region in each time frame produced a 1D curve of the type shown in Figure 2. Local maxima of the curve were selected by comparing the values at adjacent locations, and ignoring smaller peaks that were not separated by at least an intermediate time point. The function *findpeaks* in MATLAB was used to do this. This procedure was repeated with the negative of the curve to identify nadir locations. The high peaks were considered the more diastolic phases, and the nadirs the systolic phases, as noted in Figure 2. Every image was then binned to near systole or near diastole, depending on if its value was closest to the nearest peak or trough. Each slice was processed separately.

### Deformable image registration

**Figure 3:**
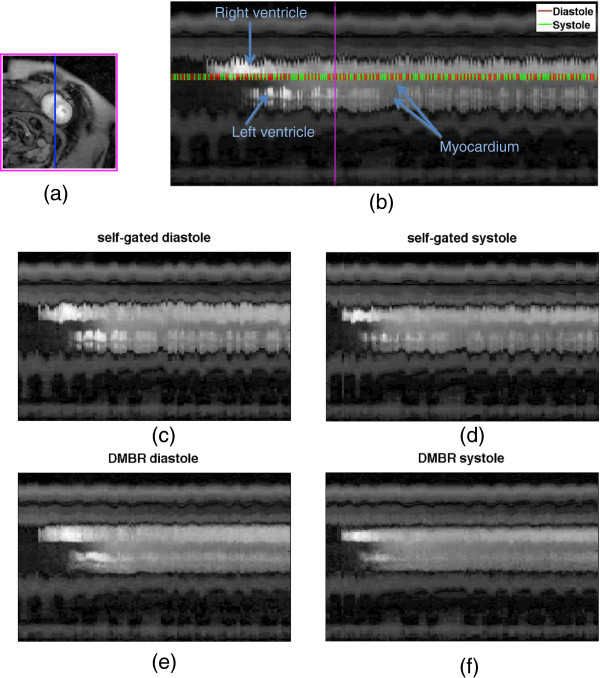
**Example of deformable registration process. (a)** Indication of the placement of the line profile that appears over time in the right. This particular frame is end-expiratory and appears in **(b)** as the pink vertical line. **(b)** Intensity profile over time for this ungated acquisition. Red lines indicate diastolic phase and the green colored lines indicate systolic phase. **(c)** Intensity line profiles after self-gating to diastole (all red lines). **(d)** After self-gating to systole. **(e** and **f)** Intensity line profiles after deformable model-based registration (DMBR).

The self-gating method was then followed by a two stage deformable image registration in order to remove residual cardiac and respiratory motion present in the near-systolic and near-diastolic images. The first stage involved creating a unique reference image for each of the time frames using a model-based approach [20,21] to account for changing contrast in the images and avoid mis-registrations. Model reference images were generated as described in [20] by choosing an input function based on a signal intensity time curve from a region of interest in the right ventricular blood pool. Multiple reference images with contrast variation similar to the underlying images but with motion suppressed were generated using a four-parameter compartment model [20]. Each acquired image was then registered to its model reference image using an automatic finite element based deformable registration. The method was based on deforming triangulated meshes assuming a linearly elastic physical model. The weighting factor for the elasticity of the model was chosen empirically for one dataset to be 4000 and was fixed to that value for all of the other patient datasets. To avoid blurring in the final registered images, a b-spline interpolation was used for the registration. Final registered images from each time frame were then interpolated in time to match the temporal resolution of the ungated acquisition using a cubic interpolation method. Figure 3 shows an example of the process and the results in one subject.

Respiratory motion did not cause very local peaks and thus typically did not affect the self-gating to systole/diastole, which permits recovery of datasets more similar to free-breathing datasets obtained with ECG gating.

Three movies showing example results from this process are provided online (Additional file 1: Movie 1, Additional file 2: Movie 2, Additional file 3: Movie 3).

### Application to diagnosis of ischemia

#### Patient selection

From August 2011 to April 2012, 8 patients who were being referred to x-ray coronary angiography or had recently undergone clinically indicated x-ray coronary angiography within the previous 2 weeks were recruited to the study at the University of Utah hospital. These patients were consecutive, in the sense that all patients imaged with the ungated approach and that had angiography results were included. Patients with a clinical history of arrhythmia were actively sought in communication to referring physicians, though this history was not a formal part of the inclusion criteria. Exclusion criteria: the presence of pacemakers or defibrillators, contraindications to adenosine (e.g. reversible airway disease, atrioventricular block), contraindication to gadolinium (allergy or GFR < 30 ml/min per 1.73 m^2^), pregnancy, claustrophobia, and the inability to lie flat for the study. Patients all confirmed that they did not have caffeine (caffeinated beverages, coffee, tea, or chocolate) for 12 hours prior to MR imaging. The study was approved by the University of Utah institutional review board and written consent from the participants was obtained.

#### Patient CMR acquisition and reconstruction

CMR was performed with the ungated sequence as described above and either a 32-phased array coil (Rapid Biomedical) or a 15-element phased array cardiac receiver coil was used, with 128–144 readout points in FOV=260-280 mm, ~2×2×8 mm pixel size. In one subject the FOV was 330 mm which gave a 2.6×2.6×8 mm pixel size. Note the actual FOV and number of samples is doubled for each ray, as is common in the readout direction. Twenty (n=6) or twenty-four (n=2) rays in 4 subsets of 5–6 rays each were acquired for each slice. The starting angle was offset in each time frame, repeating every four time frames [16]. Instead of this interleaved type of acquisition, the golden ratio increment was used in the last five subjects. That is, every subsequent ray was incremented by 111.25°, even between time frames, so that no angles were repeated. Instead of a composite saturation pulse with three 90s, a hybrid pulse train [16] was used for the saturation in the last 3 patients.

Stress perfusion CMR was performed with pharmacological hyperemia (after 2.5-3 min of adenosine infusion at a dose of 140 μg/kg/min), using 0.075 mmol/kg gadoteridol (ProHance, Bracco Diagnostics Inc., Princeton, NJ) injected into a peripheral vein with a power injector at 5 mL/s followed by a 25 mL saline flush. Rest perfusion was performed 10–20 minutes after stress with an additional bolus of 0.075 mmol/kg gadoteridol. The patient was instructed to breath shallowly during both stress and rest imaging. Late gadolinium enhancement (LGE) images were acquired approximately 10–20 min after rest perfusion imaging in long-axis and several short axis planes using a phase-sensitive inversion-recovery gradient echo sequence. The LGE short axis slices approximately matched the slice positions acquired in perfusion imaging.

The perfusion images were reconstructed with the iterative methods described above. The reconstructed ungated images were saved for review. Further processing was performed to create systolic and diastolic gated series of images using the self-gating and deformable registration methods described above.

#### Image quality

Analysis of image quality and artifacts was performed by 3 experienced observers (B.W., A.S., and C.M., with over 30 years combined experience). Image quality and artifact assessment was performed on the unprocessed ungated images, the near-systolic phase, and the near-diastolic phase images. The image quality grading scale was numeric (5–1, highest quality to lowest quality) and the artifact grading score was numeric (5–1, least artifact to most artifact). All ungated, systolic, and diastolic images were pooled to give 24 image sets each with 4–5 slices and both stress and rest. Each of these image sets were presented in a blinded fashion in a random order. Readers also commented on the type of artifact seen if artifact was present.

#### Determination of ischemia and infarct

Analysis for perfusion deficits was performed by the same 3 observers on each set of images (ungated perfusion, self-gated in systole, and self-gated in diastole). Perfusion defects were assessed by visual comparison of short axis slices in stress and rest side-by side using a 16 segment model (20). Grading of these segments was as follows: 0 = normal, 1 =equivocal but probably normal, 2 = subendocardial or full-thickness ischemia, and 3 = fixed perfusion defect that is likely infarct. The readers were blinded to all patient clinical data and to the LGE images from the study. Blinding readers to the LGE helped avoid bias to the interpretation of the perfusion datasets but would not be done in clinical practice.

### Coronary X-ray angiography

Coronary x-ray angiography was performed on 7 of the 8 patients and was evaluated in the patient’s routine clinical care by interventional cardiologists performing the patients angiograms blinded to the clinical scenarios and perfusion CMR findings. The degree of CAD was reported in the patient’s record and defined by the presence of 70% luminal narrowing measured in at least 2 orthogonal planes present in ≥1 of the three main coronary arteries or in a major side branch of ≥2 mm diameter. In the eighth patient, a positive LGE scan was used in place of the coronary x-ray angiography.

### Statistical analysis

For image quality comparison, each individual patient's image quality was averaged over the three blinded readers and the mean ± standard deviation reported. All statistical analyses were performed using STATA 11 (StataCorp, College Station, Texas). A one-way ANOVA was used to compare if any of the three image reconstruction groups (ungated, self-gated to systole, self-gated to diastole) were significantly different from each other in quality. Paired t-tests were implemented to compare the mean image quality scores between stress and rest for each of the reconstruction categories (ungated, self-gated to systole, self-gated to diastole). Diagnostic accuracy and its confidence limits were calculated by pooling the three reviewers and considering each diagnosis. For all statistical comparisons, a P value of <0.05 was considered to be statistically significant.

## Results

### Patient demographics

Of the eight patients, six had a history of atrial fibrillation, and three were in atrial fibrillation during CMR. Seven of the patients had x-ray angiograms; one did not proceed for coronary catheterization and we used a positive infarct on late gadolinium imaging as an endpoint instead of catheterization. Of the eight subjects, four were normal, two had fixed perfusion defects that correlated with infarct on LGE, and two had reversible perfusion defects consistent with obstructive coronary artery disease.

### Image quality

**Figure 4:**
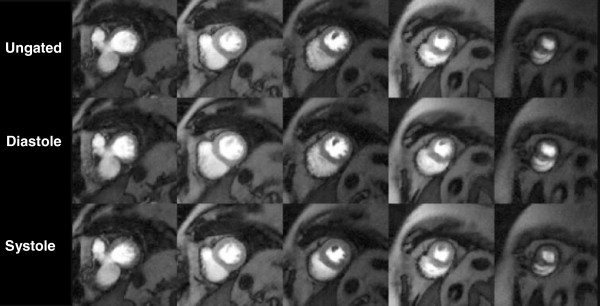
**Image quality comparison of short-axis slices from the raw ungated acquisition and the same post-processed images self-gated to systole and diastole using the methods described in the text.** This patient was in atrial fibrillation during the study. See Additional file 1: Movie1, which is the same patient.

Both the ungated and self-gated images provided good image quality. Figure 4 and online Additional file 1: Movie 1 show sets of frames from basal to apical short axis planes from a study reconstructed with the three methods (ungated, self-gated to systole, and self-gated to diastole). Limitations of the two stage model-based deformable registration can be seen with the large rapid changes in the RV such as in the most basal slice of Additional file 1: Movie 1.

The three groups (ungated, self-gated to systole, and self-gated to diastole) were not significantly different for image quality or artifact (Table 1). There were also no significant differences between image quality or artifact in the stress versus rest images. Artifacts reported included streaking and septal darkening that was transmural and present prior to contrast administration. There was no dark rim artifact seen in any of the cases. Pooled image quality scores were similar between patients in atrial fibrillation and normal sinus rhythm (3.6±0.7 vs. 3.3±0.5). Additional file 1: Movie 1 is from a patient in atrial fibrillation at the time of the scan.

**Table 1:** **Average image quality and artifact scores of ungated**, **self**-**gated systole**, **and self**-**gated diastole datasets as determined by three blinded reviewers**

**Image type**	**Image quality(Stress)**	**Artifact(Stress)**	**Image quality(Rest)**	**Artifact(Rest)**
	**Mean**±**SD**	**Mean**±**SD**	**Mean**±**SD**	**Mean**±**SD**
Ungated	3.4±0.6	3.6±0.8	3.5±0.4	3.7±0.7
Systole	3.2±0.7	3.3±0.8	3.2±0.7	3.3±0.8
Diastole	3.4±0.6	3.7±0.8	3.4±0.6	3.7±0.8

### Correlation with X-ray angiography

**Figure 5:**
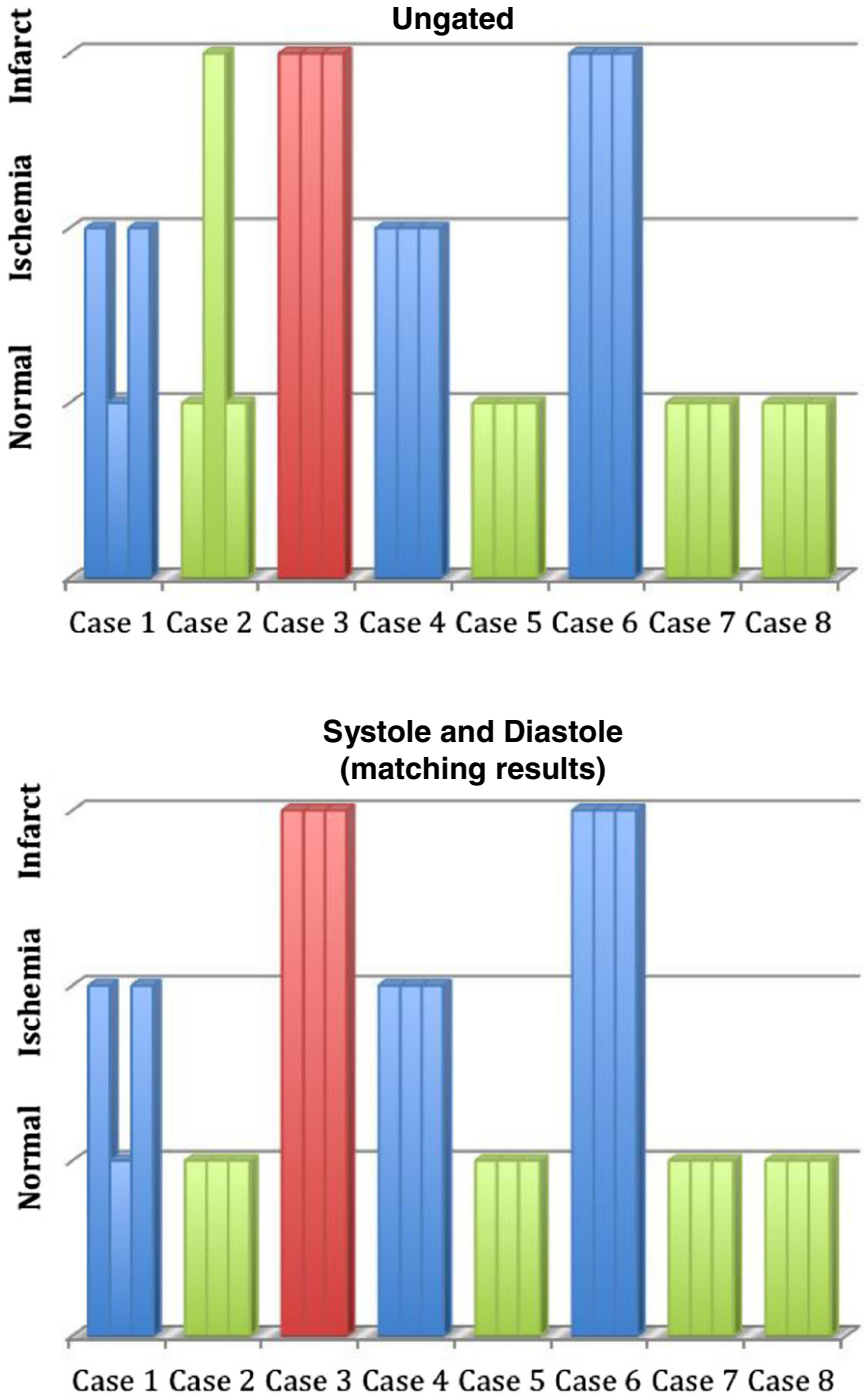
**Catheterization results and diagnosis (normal, ischemic, or infarct) from each of the three readers for each case.** Cases with positive obstructive disease on catheterization are depicted in blue, cases with no obstructive disease on catheterization are depicted in green, and the case without catheterization but with infarct on LGE is depicted in red. Case 6 had both obstructive disease on catheterization and infarct on LGE.

**Figure 6:**
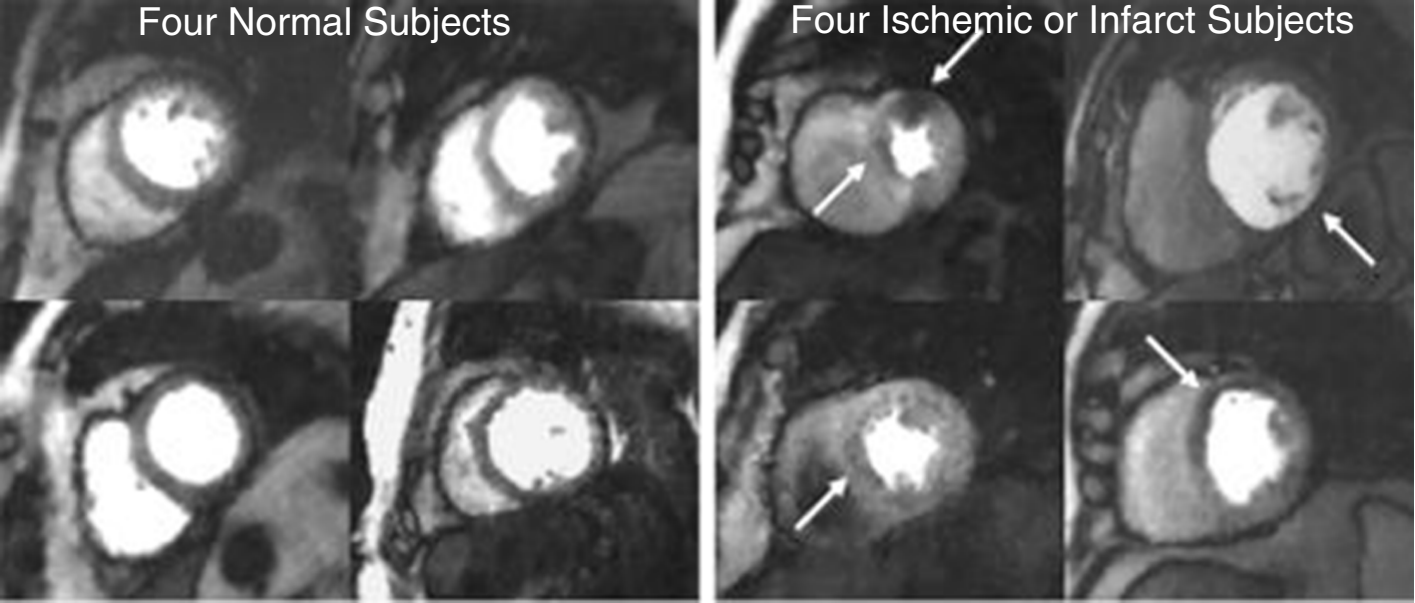
**One time frame of one slice from each of the 8 patients - ungated stress perfusion imaging.** The four images on the left are from patients with normal x-ray angiography results. The four images on the right depict reversible ischemia in two images and fixed perfusion defects consistent with infarction in the far right two images.

**Figure 7:**
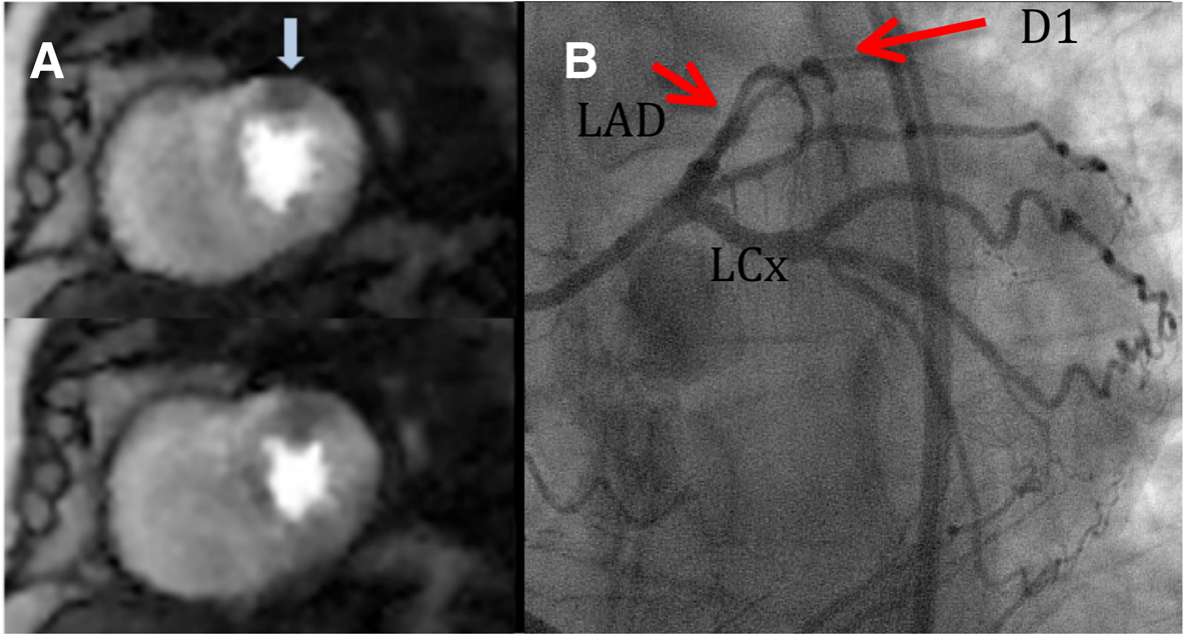
**Self-gated stress perfusion with images depicting a diastolic and a systolic cardiac phase (A) and coronary angiogram (B).** The perfusion images show a mid anterior wall defect (thick arrow). The angiogram shows severe in-stent stenosis in the first large diagonal (arrow) and chronic total occlusion of mid LAD.

Pooled results from 3 blinded readers gave a sensitivity of 0.92 (CI 0.65-0.99) and specificity of 0.92 (CI 0.65-0.99) for the detection of CAD when the ungated images were read. When either the near-systolic images or near-diastolic images were used, the sensitivity remained the same as for the ungated case. The specificity, however, changed to 1 (CI 0.76-1) as one reader called an infarct in one ungated dataset but not in the near-systole or near-diastole datasets. The diagnoses from all of the readers are portrayed in Figure 5. If each subject’s diagnosis had been determined instead by averaging the readers’ scores to concensus, then the sensitivity and specificity would have both been 1. Figure 6 shows one time frame of a short axis slice from each patient to illustrate the image quality in both normal and disease cases. Figure 7 shows an example of concordant perfusion and x-ray angiogram results.

## Discussion

We present a novel perfusion CMR method acquired without the need for ECG gating due to the rapidity of the acquisition. After iterative reconstruction of the undersampled data, the perfusion images can be viewed in an ungated format or retrospectively sorted into self-gated perfusion datasets. When compared to conventional ECG-gated perfusion sequences, this approach is efficient as it ensures that maximal information is obtained during the relatively brief first pass of contrast. That is, even with careful set-up of the conventional method, it is difficult to choose the number of slices to acquire each heartbeat. Choosing too many slices means that some triggers will be skipped, and choosing too few slices means that for portions of each heartbeat the scanner will be idle. And if the HR changes even slightly this can lead to acquiring only every other beat, which is only 50% efficient even when the ECG triggering is perfect. This ungated approach is easy to use since a good ECG signal is not required, and the number of slices is fixed prior to acquisition. The approach is also ideal for patients with non-sinus rhythm that need a noninvasive test for coronary artery disease as many of these patients have previously been excluded from MR and CT perfusion imaging due to concerns over adequate perfusion imaging.

Somewhat surprisingly, reconstruction methods with spatial and temporal TV constraints that we have used previously gave image quality similar to what we obtain with gated cardiac perfusion data with respiratory motion. We expected that it would be critical to include motion compensation within the reconstruction, but the algorithm was robust to cardiac as well as respiratory motion and produced diagnostic quality images. This is likely due to radial sampling of the data and using an L1 norm based temporal constraint that exploits temporal sparsity and does not penalize rapid changes as much as an L2 norm constraint [22]. However, incorporation of motion compensation methods within the reconstruction may provide improved image quality [23,24].

The “real-time” acquisition concept has been used for many CMR applications, and Guttman et al. illustrated a single slice perfusion study with data sharing that provided wall motion and first pass perfusion simultaneously [25]. The work here is the first to use highly accelerated acquisitions with advanced reconstruction methods and apply the real-time concept to multi-slice myocardial perfusion imaging. Likewise, self-gating is well-known, although it has not previously been used for cardiac perfusion. Larson et al. described several self-gating methods for cine CMR [6], including a region of interest sum as used here and methods such as the use of a reference frame for self-gating cine data [6]. We experimented with the reference frame method in which a correlation value between the reference frame and each other frame was computed as in Larson et al. [26]. This method was dependent on the choice of reference frame and was less reliable than the region sum method used here. Others have developed and validated self-gating approaches for cine CMR [27], and have included respiratory motion as well [18,19,26], although these methods need not consider contrast changes over time.

While this work uses a 2D ungated sequence with a saturation pulse, we recently considered doing away with the saturation pulse and performing ungated steady-state acquisitions with a 3D readout [28], since a primary reason for using a saturation pulse is to reset the magnetization after any gating loss or arrhythmia. The current work here uses saturation recovery because it is a much more tried and proven method and is universally used for perfusion studies. More work is needed to determine if the steady-state method in [28] will be useful in practice.

In application to a small population of patients with suspected or established coronary artery disease, perfusion CMR with an ungated acquisition has a high diagnostic accuracy when coronary x-ray angiography is considered as the reference. Little difference was seen in the visual assessment of perfusion defects or perception of image quality between either reading off the ungated acquisition or from retrospectively self-gating the images into near-systole or near-diastole image sets. The finding of similar diagnostic accuracy at systole or diastole is consistent with a recent study by Motwani et al. [29], although that was with the acquisition of a single slice. It is possible that having both near-systolic and near-diastolic data will improve diagnostic accuracy and this awaits larger studies. Wall motion at vasodilatory stress could be obtained as well, which may be useful diagnostically [30].

One limitation of the self-gated approach is that the deformable registration could possibly make perfusion defects more difficult to detect, or could introduce artifacts. We have not noticed such masking of perfusion defects in this work, but more studies are needed.

In the current study, there was little change in the quality ratings of the scans when the reading was done from the ungated sequence of images. All of the studies were acquired during shallow breathing and the reconstruction and reading were seemingly not affected adversely by this approach. Dark rim, an artifact that frequently complicates the interpretation of perfusion CMR, was not seen in any of our acquisitions. This is typical in our experience with rapid radial acquisitions, and is likely due in part to the rapid readout such that cardiac motion is not an issue. The lack of dark rim is also likely partly due to the good spatial resolution in all directions inherent to the radial acquisition, which does not use phase encoding. Often the phase encoding direction has worse spatial resolution and that can contribute to dark rim artifact [31].

Image quality was preserved in the patients in atrial fibrillation at the time of the study. This is important since there is a pressing need in the atrial fibrillation population for reliable noninvasive methods for the detection of obstructive CAD. Atrial fibrillation is becoming more widespread and has a high association with CAD [32]. Currently many of these AF patients are referred directly to an invasive x-ray angiogram as other noninvasive tests such as dobutamine echo, coronary CTA, and nuclear perfusion testing have not been good alternatives for noninvasive testing in this subset of patents [33]. If an ungated or self-gated approach has a diagnostic and image quality that in an arrhythmia is comparable to sinus patients, MR may be able to fill this noninvasive imaging void.

The main limitation of the clinical portion of this study is the small sample size. The goal of the study was not to prove diagnostic accuracy, but to demonstrate initial development of the novel ungated and self-gated approaches and to perform a first evaluation. Since these initial results show promise, the diagnostic accuracy of the ungated and self-gated approaches will need to be validated in larger clinical studies.

## Conclusion

This study shows that in this small population of patients with suspected or established coronary artery disease, perfusion CMR with a rapid, ungated acquisition has a high diagnostic accuracy when coronary x-ray angiography is considered as the reference. Little difference was seen in the visual assessment of perfusion defects or in the perception of image quality between either reading off the ungated acquisition or from retrospectively self-gating the images into near-systole or near-diastole image sets. Image quality was also preserved in the patients in atrial fibrillation at the time of the study. These findings suggest that a self-gated or ungated perfusion CMR approach could contribute to making myocardial perfusion CMR simpler and more accessible by allowing for greater ease of imaging patients. The approach may be of particular value in patients with poor gating or significant arrhythmias.

## Abbreviations

AF: Atrial fibrillation; CAD: Coronary artery disease; CMR: Cardiovascular magnetic resonance; CTA: Computed tomography angiography; ECG: Electrocardiograph; GFR: Glomerular filtration rate; LAD: Left anterior descending; LGE: Late gadolinium enhancement; LV: Left ventricle; RV: Right ventricle.

## Competing interests

The authors declare that they have no competing interests.

## Authors’ contributions

All authors were involved in guaranteeing the integrity of the study. AH was involved with study design, all of the data acquisition, the data interpretation, and performed the initial review of the literature and manuscript drafting. ED and GA designed the sequence and developed the image reconstruction and post-processing. DK contributed the custom saturation pulse for the sequence. ED, CM, BW, NM, AS were involved in the study concepts/study design, data acquisition and data analysis/interpretation. KD performed the statistical analyses. All authors were involved with manuscript revising and editing. All authors read and approved the final manuscript.

## Supplementary Material

Additional file 1: Movie 1 Stress study of normal, which corresponds to Figure 4. Top row: ungated, middle row: self-gated to near diastole. Bottom row: self-gated to near systole. The bottom two rows were interpolated in time to match the number of ungated frames. Both quality and artifact scores were 4.3 for this study for ungated and self-gated. Click here for file

Additional file 2: Movie 2 Stress study of subject with ischemia. The format is the same as in Additional file 1: Movie 1. Image quality score was 3.3 for ungated and both self-gated image sets. Artifact scores were 3.3, 4, and 3.7 for the ungated, near-diastole, and near-systole (top to bottom rows). Click here for file

Additional file 3: Movie 3 Rest study of the same subject as in Additional file 2: Movie 2. Image quality score was 3.3 for all three rows, and the artifact scores were 3.3, 4, and 3.7 for the ungated, near-diastole, and near-systole (top to bottom rows). Click here for file
